# Nipping Adipocyte Inflammation in the Bud

**DOI:** 10.20900/immunometab20210012

**Published:** 2021-02-14

**Authors:** Michael J. Griffin

**Affiliations:** Sam Houston State University, College of Osteopathic Medicine, 975 City Central Avenue, Conroe, TX, 77304 USA

**Keywords:** adipose inflammation, Ebf1, toll-like receptor, 3T3-L1

## Abstract

Adipose tissue inflammation continues to represent a significant area of research in immunometabolism. We have identified a transcription factor, EBF1, which crucially regulates the expression of numerous inflammatory loci in adipocytes. However, EBF1 appears to do so without physically binding to these inflammatory genes. Our research is currently focused on understanding this discrepancy, and we believe that future findings could pave the road for drug development aimed to block adipose inflammation at its source.

## INTRODUCTION

Although it has been well-established that obesity is associated with chronic inflammation in adipose tissue, the molecular events that trigger the initial inflammatory response in adipocytes remain mostly unexplored. The vast majority of the work in the adipose inflammation field has focused on the comings and goings of the associated immune cells, especially M1-activated macrophages. But what triggers the invasion and activation of immune cells, to begin with? I believe that the adipocytes themselves are anything but a passive player in adipose tissue inflammation and that the process involves significant changes in transcriptional reprogramming in fat. I liken the initiation of inflammation in adipose tissue to starting a jet engine: on the ground, an Auxiliary Power Unit (APU, the adipocytes in this analogy) gets the main turbofan engine (the immune cells) rotating until the jet engines become self-sustaining. When that point is achieved, the pilot can power down the APU. An APU failure will mean that the engines cannot be turned on from a cold start. But most studies have focused on reverse-engineering the turbofan engines, not the APU of inflammation in the adipose biology field.

To wit, while genetic studies of adipose inflammation are prevalent, very few studies have examined tissue-specific/loss-of-function of individual genes in adipocytes in vivo. In 2006, Shi et al. famously demonstrated that mice with a germline deficiency of *Tlr4*—which plays a key role in TLR (Toll-Like Receptor) signaling in adipocytes—display reduced insulin resistance, a telltale sign that underlying inflammation had been stymied [1]. However, many tissues avidly express *Tlr4*, especially macrophages, impossibly confounding any interpretation of the specific effect of this receptor protein’s contribution to the inflammatory process in adipocytes. Since then, many other studies have demonstrated the effects of *Tlr4* deficiency and other inflammatory players on adipose tissue and adipocytes, but most of these studies used either whole-body knockouts or deletion of genes in cells other than adipocytes. Thus, I believe that the field has largely overlooked the inner workings of the adipocyte as an inflammatory pilot light, and my lab currently seeks to understand how the process is regulated at the transcriptional level.

## THE CURRENT DILEMMA

My entry into the adipose inflammation field came serendipitously. In 2013 during my postdoctoral work in the laboratory of Evan Rosen at the Beth Israel Deaconess Medical Center in Boston, we stumbled upon an exciting discovery in the adipose biology field. At the time, we were trying to unbiasedly determine the function of a lymphopoietic transcription factor, Early B-Cell Factor-1 (Ebf1), in the mature adipocyte. (Note: in mice and man, olfactory nerves and B-cells are the only other cell types that express significant amounts of this gene.) Transcriptomic analyses of Ebf1-deficient adipocytes (EDAs) indicated that Ebf1 regulates several different signaling pathways, with TLR signaling standing out as the most significant “hit” [2]. The basal (uninduced) expression of several critical inflammatory chemokines (e.g., *Ccl2, Ccl5, Cxcl10*) was severely reduced, as were genes encoding several upstream signaling components in this pathway (see Table 1 for a listing of selected inflammatory genes whose expression was significantly altered in EDAs). Further investigation revealed that lipopolysaccharide (LPS)-stimulated secretion of Ccl5 (RANTES), Cxcl10, and Il6, Erk phosphorylation, and lipolysis were largely taken out of action. However, a stunning quandary arose when it became clear that Ebf1 protein does not seem to physically occupy DNA near or within the gene bodies of almost all of the TLR signaling and chemokine loci whose expression was reduced in EDAs. This finding was supported by data from the genome-wide ChIP-seq analysis using a monoclonal Ebf1 antibody in non-induced resting mature adipocytes and stood in stark contrast to the situation with insulin signaling: the reduction in Ebf1 protein also impaired the expression of several major components of the core insulin signaling pathway (*Irs1*, *Pik3r1*, *Akt2*, etc.), and ChIP-seq data unambiguously showed high occupancy of these loci by Ebf1. Yet, the reduction of TLR-related genes in EDAs was statistically more significant than that of the insulin signaling pathway. This observation called into question the molecular mechanisms through which Ebf1 regulates inflammatory gene expression in adipocytes. This quandary remains an intensive focus of current work in my lab.

**Figure 1.**
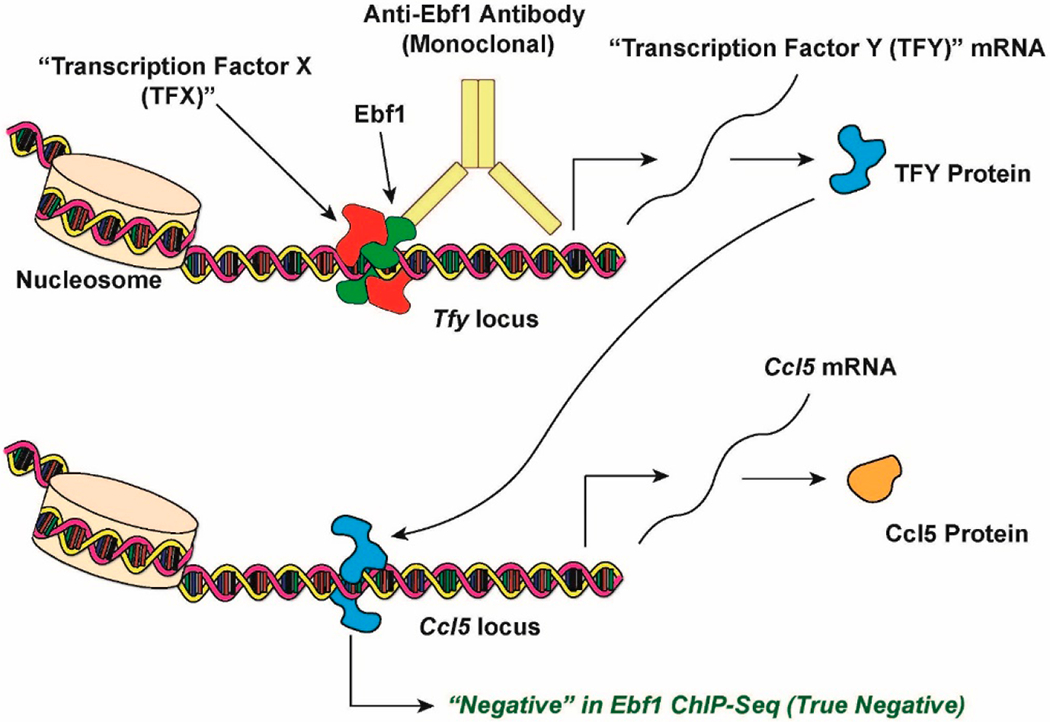
Proposed Explanation for Previous Negative Results In ChIP-seq. See main text for details.

**Figure 2.**
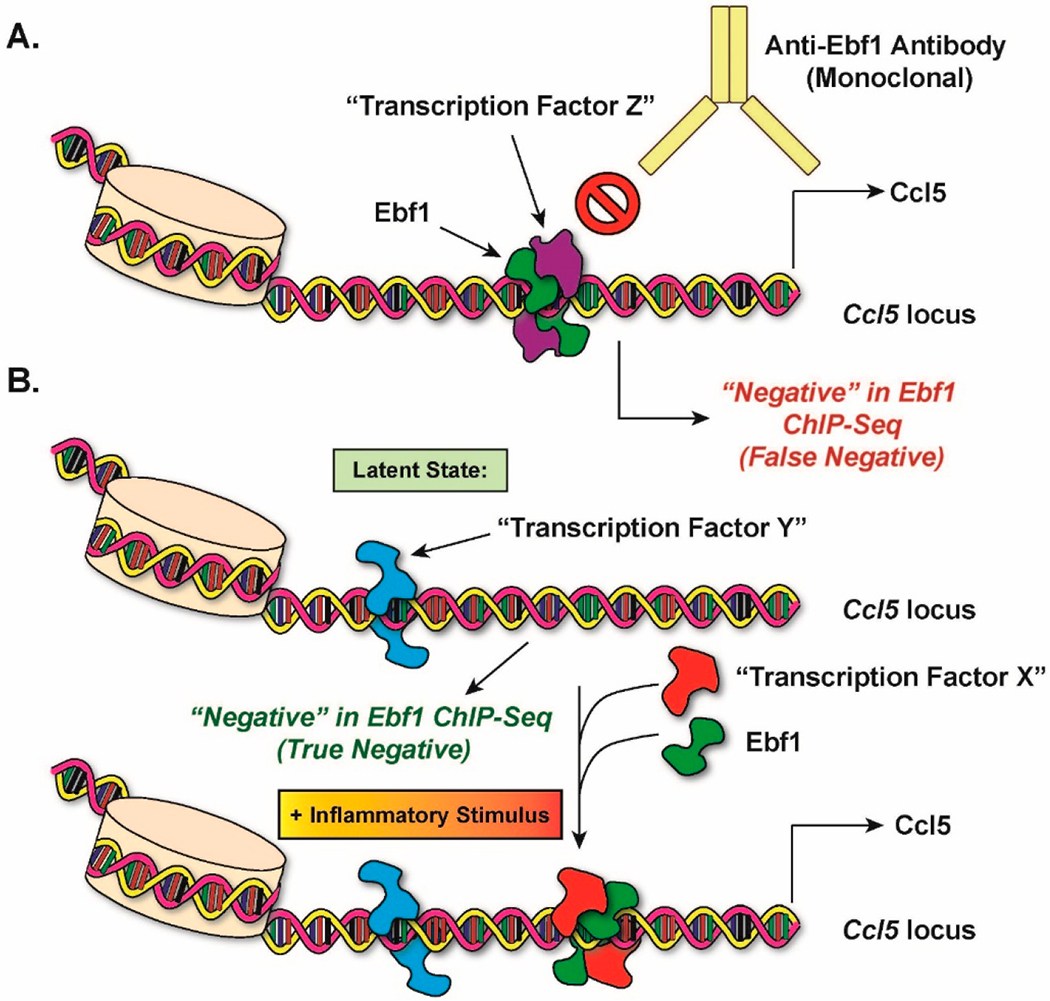
Alternative Explanation for Previous Negative Results in Ebf1 ChIP-seq. (**A**) Model for a scenario in which Ebf1 is, in fact, bound to the promoters of inflammatory genes, but was not detectable in our original ChIP-seq assay (false negative). (**B**) Model for a scenario in which Ebf1 is induced to bind to promoters of inflammatory genes following an inflammatory stimulus. See main text for details.

In the meantime, the most parsimonious explanation for our “spooky action at a distance” dilemma (Ebf1 regulates inflammatory genes without *apparently* binding to them) is that Ebf1 must be working with or through one or more “unknown” transcription factors (Figures 1 and 2). At present, I believe that two possible models could be used to explain our previous results indicating that Ebf1 regulates several inflammatory loci in adipocytes without (apparently) binding to them (Figures 1 and 2). One explanation for this observation is that Ebf1 regulates these loci in a *trans*-configuration: using the *Ccl5* locus as an example model, Ebf1 binds to the promoter (or an enhancer) of an unknown transcription factor, “Transcription Factor Y”, perhaps in cooperation with “Transcription Factor X”. Transcription factor Y, in turn, binds to the promoter (or an enhancer) associated with the *Ccl5* locus directly, and stimulates its transcription. This model could explain the apparent discrepancy observed in my previous study: knocking down Ebf1 would, in turn, result in decreased protein levels of Transcription Factor Y, thereby reducing Ccl5 levels as well. Since Ebf1 is not directly bond to the *Ccl5* promoter (or any associated enhancers) in this model, my original ChIP-seq results would have been correct and valid.

In an alternative model, I believe that my previous ChIP-seq results with Ebf1 [2] could have represented “false negatives” with respect to Ebf1 binding to the inflammatory loci whose expression was significantly impaired in Ebf1-deficient adipocytes (Figure 2). In panel (A), I propose that Ebf1 interacts with an unknown “Transcription Factor Z” at the *Ccl5* locus, but in a mutually exclusive configuration with the monoclonal Ebf1 antibody used for the ChIP pulldowns in my study. In other words, Transcription Factor Z could be interacting with Ebf1 at a subset of Ebf1-regulated loci in such a way that prevents the antibody from accessing its Ebf1 epitope during the immunoprecipitation reactions. The ChIP-seq would thus present a false negative at the *Ccl5* locus. Another possibility (B) is that the binding of Ebf1 to the *Ccl5* locus depends on the prior binding of an inflammatory stimulus-inducible transcription factor. In this model, the lack of apparent Ebf1 binding to the *Ccl5* locus could be explained by the fact that I conducted my previous ChIP-seq experiment under basal, non-stimulated conditions. It is possible that were we to repeat the experiment with a stimulus (e.g., LPS), an unknown transcription factor would bind to the promoter in an ordered assembly followed by Ebf1. This configuration may or may not score positive in ChIP-seq, depending on the accessibility of the antibody. Although it would not necessarily affect the ChIP-seq results with regard to Ebf1, this model may involve the constitutive binding of a third transcription factor that helps keep the chromatin in an open state.

## PHYSIOLOGY OF EBF1 IN MOUSE AND MAN

Studies of whole-body *Ebf1*^−/−^ mice have confounded interpretations of the role of this protein in adipocyte inflammation in vivo. On a pure C57Bl/6 background, many *Ebf1*^−/−^ animals die in utero or shortly after birth [3,4]. On a mixed background, *Ebf1*^−/−^ mice are severely lipodystrophic [4,5]. Such a finding was entirely predictable, as several previous studies had indicated the indispensable role of Ebf1 in adipocyte differentiation in cell culture models [6–8]. However, the profound lack of subcutaneous adipose tissue in the *Ebf1*^−/−^ animals precludes any meaningful analysis of the role of this protein in inflammation in White Adipose Tissue (WAT). Perhaps the most striking finding of the paper published by Fretz et al. in 2010 was that these animals were mildly-hypoglycemic and relatively insulin-sensitive [5]; most other genetically-engineered lipodystrophic mouse models have presented with fasting hyperglycemia and severe insulin resistance. The fasting hypoglycemia persisted even in the face of elevated glucagon levels in the *Ebf1*^−/−^ animals; for the most part, this very peculiar metabolic phenotype remains mechanistically unexplained.

Most of the work in the field of Ebf1 in adipose biology has focused on its role in adipocyte differentiation. Several in vitro studies have unequivocally demonstrated that Ebf1 is necessary, but not sufficient, to drive adipogenesis in the 3T3-L1 or 3T3 model cell lines [6,7,9]. The primary mechanism through which Ebf1 serves this role is through binding to the promoters of C/EBPα and PPAR-γ, two essential players in the process [7]. Ebf1 may also be involved in the commitment process. Its activity is inhibited by the zinc-finger protein Zfp521, which prevents Ebf1 from binding to key adipogenic loci in preadipocytes [9]. Of note, Zfp521, to my knowledge, is one notable member on a very short list of Ebf1 interaction partners in adipocytes or their precursors. (A few groups have identified other Ebf1-interacting proteins, including CBP/p300 [10], but whether these interactions occur in adipocytes was not investigated). Although I believe that the role of Ebf1 in adipocyte inflammation is a completely separate process than its role in differentiation, the two processes become intertwined when attempting to deconvolute human EBF1 studies: innately low *EBF1* expression or activity will likely impair adipogenesis, which will almost certainly lead to metabolic disease; but higher or just “normal” levels may permissively foment WAT inflammation, also leading to insulin resistance and metabolic disease.

Another potential protein interaction partner for Ebf1 in adipocytes is its orthologous cousin, Ebf2. (An early study demonstrated that Ebf1 can form heterodimers with Ebf2, although this was shown only in the in vitro context of electrophoretic gel shift assays [11]). In a recent study [12], Angueira et al. developed adipocyte-specific *Ebf1* (*Ebf1^ΔAdipoq^*) and *Ebf2* (*Ebf2^ΔAdipoq^*) knockout animals using the same driver that we intend to use for our studies (Cre recombinase driven by the *Adipoq* promoter, see below). The *Ebf2^ΔAdipoq^* mice presented with severe derangements in BAT function and thermogenesis, with significant decreases in expression of UCP1. Combined deletion of *Ebf1* and *Ebf2* had the greatest impact on thermogenesis in BAT, suggesting that the two proteins normally work together to promote heat dissipation in thermogenic tissue. Deletion of *Ebf1* alone, however, had minimal impact on thermogenesis or BAT function, suggesting that Ebf1 is dispensable for the regulation of thermogenic genes in BAT and that Ebf2 might form homodimers with itself at key enhancers as a compensatory measure in the *Ebf1^ΔAdipoq^* animals (I should like to note that when *Ebf1* was “knocked down” in 3T3-L1 adipocytes in my hands with two different shEbf1 constructs, the expression of Ebf2 increased [2]. However, basal *Ebf2* expression in adipocytes in vitro—and in WAT—is an order of magnitude less than that of *Ebf1* [13]). Thus, any therapeutic intervention in humans that reduces expression of *EBF1*, or reduces EBF1 protein function in mature WAT, should have minimal impact on the generally desirable process of fomenting thermogenesis or promoting the development of BAT. The effect on adipogenesis, however, would be a different story.

Prior to my work in 2013, only one other group had examined gene regulation by Ebf1 in mature 3T3-L1 adipocytes, and that was not by design. In a 2004 study [14], Dowell & Cooke serendipitously discovered—using a one-hybrid screen—that Ebf1 can bind to a putative negative regulatory element in the *Slc2a4* gene (encoding the glucose transporter Glut4) in competition with another transcription factor, NF1. This binding was detected in mature adipocytes as well as WAT and several other moue tissues. The authors proposed that Ebf1 mediates insulin repression of *Slc2a4* through this element (*“glut4 IRE”*) in adipocytes. This was supported by Electrophoretic Mobility Gel Shift (EMSA) assay data and mutational analysis indicating that mutation of this DNA element abolished repression of *Slc2a4* by insulin. However, in my hands, there was no ChIP-seq peak corresponding to the specific *glut4 IRE* site identified by Dowell & Cooke [2]. This suggests that Ebf1 does not occupy the Dowell & Cooke site in live cells, that insulin treatment is required to drive Ebf1 binding to this site, or that my results simply represented a false negative. In the transcription factor field at large, this discrepancy also highlights the importance of distinguishing between what *can* happen in vitro vs what actually *does* happen in vivo. In any case, my results functionally indicated that while basal expression of *Slc2a4* was unaltered in untreated EDAs, insulin-stimulated glucose transport was significantly impaired [2]. We did not examine the expression of *Slc2a4* in insulin-treated EDAs, an experiment worth considering for future studies. I will be quite curious to see what happens to expression of *Slc2a4* in our *Fat-Specific Ebf1 Knockout (FEBKO)* animals (discussed in a later section).

## EVIDENCE AGAINST A ROLE FOR EBF1 IN ADIPOCYTE INFLAMMATION?

A few key pieces of data examining the role of EBF1 in human obesity seem ostensibly inconsistent with my hypothesis that EBF1 permissively promotes a normal inflammatory response in adipocytes, perhaps even arguing against it. In one 2015 study, EBF1 activity in adipose tissue was inversely correlated with waist circumference, BMI, and adipose morphology [15]. Likewise, in a more recent discovery-driven study, the expression of no fewer than ten anti-*EBF1* miRNAs was directly correlated with BMI/WAT hypertrophy in humans [16]. Similarly, Gao et al. found that low *EBF1* expression was correlated with adipocyte hypertrophy in humans (normally associated with adipose inflammation), and it was suggested that this hypertrophy occurred mainly on account of impaired isoproterenol-stimulated lipolysis [17]. Finally, while *Ebf1*^−/−^ animals have significantly diminished WAT mass, the adipocytes that do survive are, on average, much larger than normal [5]. Taken together, these data seem to directly contradict my hypothesis that EBF1 promotes normal adipose inflammation in obesity (since several studies have confirmed that *EBF1* or *Ebf1* expression is lower in larger—and presumably more inflammation-prone-fat cells). In any case, it would appear that there is a post-differentiation process occurring in adipocytes that leads to downregulated expression in larger adipocytes. If congenitally-determined low *EBF1* expression *per se* (possibly due to the presence of expression-influencing SNPs) was the originating factor, some adipocytes likely would not exist to begin with (a molecular equivalent of the “Grandfather Paradox”). However, that process seems to be regulated in lock-step with mature adipocyte cell size since smaller adipocytes from lean individuals boast plenty of *EBF1* expression.

**Figure 3.**
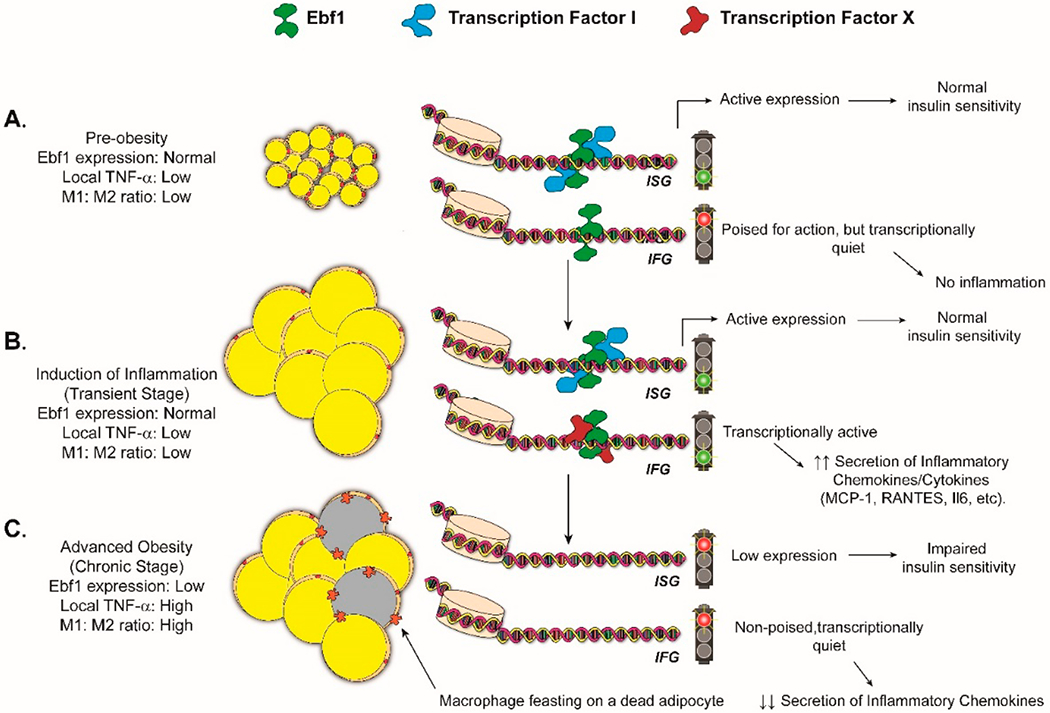
Poised for Action: Proposal for How EBF1 Plays Into Adipocyte Inflammation. (**A**) In individuals with “lean”, insulin-sensitive adipocytes, EBF1 levels reach relatively high levels in a steady state. mRNAs encoding Insulin-Signaling Genes (ISGs), such as **PIK3R1** and **AKT2**, are directly bound by EBF1 and contribute to the metabolic health of adipose tissue. (This may or may not involve the interaction of EBF1 with other transcription factors.) EBF1 may or may not be bound to enhancers associated with Inflammatory Genes (IFNs, see Figures 1 and 2) in the latent state, but transcription is maintained at only basal levels since other inflammatory transcription factors are “missing”. (**B**) At some point, one or more acute inflammatory stimuli (hypoxia, LPS, etc.) trigger(s) the activation of inflammatory transcription factors (e.g., “Transcription Factor X,”) which work with EBF1 to drive high expression of chemokines (**CCL2**, **CCL5**, **CXCL10**, etc.). This serves to recruit a battery of immune cells, most notably M1-macrophages. The macrophages secrete large amounts of TNF-α, which acts to downregulate **EBF1** expression in adipocytes (**C**). Surviving adipocytes would thus assume a quiescent state for the duration of the chronic inflammatory episode, in which neither ISGs nor IFNs are highly expressed. (Note that while we have depicted the enhancer chromatin as remaining open here, it is quite possible that it would assume a closed state in the absence of EBF1).

Striking another blow to my hypothesis, Gao et al. demonstrated in 2014 that Tumor Necrosis Factor (TNF-α)—the undisputed king of inflammatory cytokines—potently represses the expression of *EBF1* in isolated human adipocytes [17]. We have also observed that TNF-α dose-dependently inhibits *Ebf1* expression in 3T3-L1 adipocytes (Hong-Diep Vo Nguyen and MJ Griffin, unpublished observations). So how do we explain these discrepancies? I believe that these observations can be explained by nothing more complicated than a “textbook” negative feedback loop: TNF-α may downregulate *EBF1* expression in adipocytes while simultaneously stimulating the activity of other inflammatory transcription factors that depend on EBF1 protein for their functions (see Figure 2, lower panel, and Figure 3). Such a scheme is the essence of my APU/jet engine analogy: once the APU has been triggered, it can later be idled as the jet engines become self-sustaining.

My proposal for how Ebf1 regulates inflammation in adipocytes is thus as follows (Figure 3). In my model, we begin arbitrarily with a population of smaller, non-inflamed, insulin-sensitive adipocytes. *EBF1* expression should, by default, remain high at this stage (barring any genetic abnormalities that limit its expression). Higher levels of EBF1 in non-inflamed, insulin-sensitive adipocytes would be consistent with the data of Gao et al. [17]; they showed higher *EBF1* expression in “lean” adipocytes than in “obese” adipocytes. Over time, some patients may experience a chronic positive energy balance. In response, adipocytes will become larger and are eventually bombarded with any number of acute inflammatory stimuli, such as excess Free Fatty Acids (FFAs), hypoxia, mechanical strain, or EPS (reviewed in [18,19]). At this stage, EBF1 is called into action, helping the cell to produce an inflammatory burst of cytokines (“APU start”), which act to draw in various immune cells, most notably M1 macrophages (“engine start”). The macrophages respond by producing large amounts of TNF-α. The TNF-α binds to receptors on adipocytes and largely shuts down the expression of *EBF1,* thus also dialing down the expression of EBF1-dependent chemokines and cytokines in a classic negative feedback loop. These adipocytes would remain indefinitely in a quiescent state, conserving energy.

At some point, if and when the inflammation resolves, *EBF1* expression might be “reset” back to normal levels (as TNF-α levels or function decline). My proposal could explain the fact that in humans with advanced obesity and larger adipocytes, *EBF1* and EBF1 expression and activity are low, respectively, in the tissue overall (as discussed earlier). There are likely several different ways in which the APU shut-down is accomplished, with low *EBF1* expression being just one of the failsafes. I should note that alterations in adipocyte lipolysis would also make a major contribution to cell size in an EBF1-dependent manner, as described by Gao et al. [17].

If correct, this model could also elegantly explain, in part, the well-known observation that TNF-α causes insulin resistance in adipocytes (reviewed by Ruan & Lodish [20]), since normal insulin signaling in these cells depends on EBF1 activity (ref. [2]; ↑ TNF-α → ↓ Ebf1 → ↓ expression of insulin signaling genes). Indeed, many of the same insulin signaling genes that are downregulated in adipocytes by TNF-α are the same ones regulated by EBF1 [21–24]. I am currently planning experiments in which recombinant Ebf1 is added back to TNF-α-treated adipocytes using a lentiviral expression vector; I expect that this manipulation will “rescue” the expression of insulin signaling and inflammatory chemokine genes. I also point out that TNF-α inhibits adipogenesis *per se* [25–29]; I believe that TNF-α-mediated downregulation of EBF1 in preadipocytes could make a major contribution to this phenomenon in inflamed adipose tissue.

**Figure 4.**
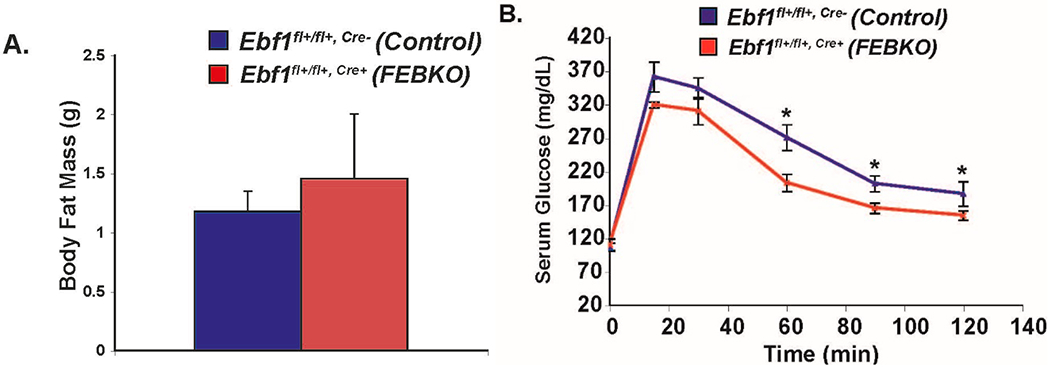
Metabolic Phenotype of FEBKO Animals. (**A**) No change in total body fat mass between control and FEBKO mice as measured by Echo-MRI scanning. (**B**) Fasting glucose tolerance in male FEBKO animals (red line) and control mice (blue line) on a high-fat diet. Following injection of 0.2 mg/kg glucose, serum glucose levels were measured at 15, 30, 60, 90, and 120 min. For control, *n* = 7; for FEBKO, *n* = 4. Student’s T-Test (A) and ANOVA (B) were used to determine significance; * p < 0.05. MJ Griffin & ED Rosen, unpublished observations.

If EBF1 regulates components of the TLR signaling pathway in humans, then investigating this further could open up avenues for rational drug design to ameliorate diet-induced adipocyte inflammation. To achieve this aim, we must pursue two lines of investigation simultaneously: (1) determine, at the molecular level, precisely how Ebf1 regulates components of the TLR signaling pathway, and (2) determine what happens in vivo when Ebf1 is lacking in mature adipocytes, and only in mature adipocytes. Work in my lab currently focuses on both. We have developed a Fat-Specific Ebf1 Knockout (FEBKO) animal model (genotype *Ebf1^fl/fl^*, *Ad-Cre*^+^, equivalent to the *Ebf1^ΔAdipoq^* animals discussed earlier), which circumvents the confounding issues of lipodystrophy as well as global *Ebf1* heterozygosity (a limitation of the Gao paper [17], in my opinion). I am pleased to report that preliminary experiments during my postdoctoral studies had demonstrated that the FEBKO animals boast no less adipose tissue than their *Ebf1^fl/fl^*, *Ad-Cre*^−^ counterparts (Figure 4A). (Apparently, the *Adipoq* promoter—used to drive expression of Cre recombinase—only becomes activated at a later stage of adipogenesis in vivo; otherwise, the animals would have presumably presented with the same lipodystrophic phenotype as *Ebf1*^−/−^ animals). The fact that these animals are not lipodystrophic, of course, makes them amenable to studies of WAT inflammation. Of note, male FEBKO mice placed on a high-fat diet appear to tolerate glucose more effectively than their wild-type counterparts (Figure 4B), at least at some time points. This observation is certainly consistent with diminished adipose and systemic inflammation. Preliminary data with the FEBKO mice during my postdoctoral years provides indirect evidence that Ebf1 is required for adipocyte inflammation in vivo and supports my current hypothesis.

I note that our results will be impossibly confounded if it turns out that animals eat less food or exhibit more physical activity—either one of these will smother inflammation in adipose tissue, with or without Ebf1. One possible solution to this dilemma will be to engineer *Ebf1^fl/fl^*, *Ad-Cre^ER^* animals, in which expression of Cre recombinase will remain latent until stimulated by Tamoxifen administration. Such a strategy would provide an opportunity to control Ebf1 inactivation temporally and acutely.

## GENETIC ABNORMALITIES IN *EBF1* MAY CONTRIBUTE TO IMMUNOMETABOLIC DISEASE

Over the last several years, evidence has accumulated that various genetic alterations in the *EBF1* locus may represent a risk factor for metabolic disease. Most of these studies appear to have been conducted by researchers previously unfamiliar with the *EBF1* gene. Several unbiased Genome-Wide Association Studies (GWAS) or targeted correlative studies have uncovered strong associations of numerous Single Nucleotide Polymorphisms (SNPs) in *EBF1* with metabolic traits or diseases, including waist circumference, BMI, Coronary Heart Disease, and blood pressure [30–38]. In one of the first of these to be reported, Singh et al. in 2015 conducted a de novo, unbiased search for SNPs in a 5800-strong multiethnic population that correlated with both metabolic disease and chronic psychosocial stress. Of the over 500,000 SNPs of interest that were identified, only five reached genome-wide statistical significance, and all five mapped to intronic regions within the *EBF1* locus. However, *EBF1* expression was not assessed [34]. The authors claimed in the title itself that *EBF1* represents a “metabolic and cardiovascular risk gene”. Another study out of China demonstrated that two SNPs in *EBF1* contribute to the risk of Coronary Artery Disease, especially in patients who drink or smoke [31]. Curiously, SNPs in *EBF1* have been identified [32] that even link to cases of Anorexia Nervosa (AN), with Fretz et al. having pointed out years earlier that the phenotype of many patients with AN—which often feature elevated levels of bone adiposity [39,40]—essentially phenocopies that of the *Ebf1*^−/−^ animals [5]. SNPs in *EBF1* have also been linked with birth weight and childhood obesity, and even gestational duration [41–45]. The association between SNPs in *EBF1* and low birth weight is intriguing. If my suspicion that these SNPs lower expression or activity of *EBF1* is correct, this would certainly be consistent with the severely runted phenotype of *Ebf1*^−/−^ animals [4]. Of note, in two recent and provocative studies from the same group, low *EBF1* mRNA levels in maternal blood were significantly associated with spontaneous premature birth [46], and higher levels of four putative *EBF1* miRNA transcripts in maternal blood were associated with a higher risk of premature birth [47].

## SUMMARY AND FUTURE WORK

The last 20 years of adipocyte biology have seen a significant paradigm shift from understanding adipocyte differentiation, mainly using 3T3-L1 adipocytes sitting in plastic dishes as a model system, to studies of inflammation in adipose tissue, largely using physiological analyses of genetically-modified mice. I believe that availing ourselves of the tools of both worlds to continue this line of investigation may eventually generate data needed for the rational design of drugs designed to ameliorate inflammation in adipose tissue. Clearly, when the Ebf1 “part” is “missing”, power is not getting to the APU of adipose inflammation. However, for any number of reasons, “floxing away” *EBF1* from adipocytes in humans—even if it were feasible at this time—seems questionable, whereas global lack of EBF1 would likely severely compromise an obese patient’s immune system, all the more undesired with the current COVID-19 pandemic. It would also likely lead to severe lipodystrophy.

To summarize, the bulk of evidence suggests that the role of EBF1 in adipose tissue may represent a double-edged sword, or “dammed if you do, dammed if you don’t” scenario: having too little of it could impair differentiation or restrict adipocyte size, leading to secondary lipid accumulation and insulin resistance in non-adipose tissues. Several prior studies have provided evidence that having little or no adipose tissue leads to metabolic disease; mouse models with congenital deficiencies of WAT are almost unequivocally insulin-resistant (reviewed in [48]). Yet, *not* having too little EBF1 in adipose tissue might permissively set the stage for a robust local inflammatory response that may or may not ever arrive in humans, depending on both genetic and lifestyle factors. As described earlier, part of the aftermath of that response may involve a semi-permanent state of TNF-α-mediated downregulated *EBF1* expression in larger, inflamed adipocytes. So, I believe that Ebf1 *initially* needs to be in place for adipocytes to mount a “normal” inflammatory response but becomes dispensable once the process has become self-sustaining through the infiltration of “professional” immune cells. In addition, I believe that EBF1 is not a *primary* driver of inflammation in adipocytes; but rather that it serves as an accessory factor to “aid and abet” other inflammatory transcription factors. We have recently identified some possible candidates for these proteins and are actively investigating them at this time.

If my hypothesis is correct, then disrupting the putative protein-protein interactions between Ebf1 and one or more of its putative transcription factor partners with small molecules or peptides could represent a viable way to “nip inflammation in the bud” in adipocytes. Since this is a highly-targeted approach, the expectation would be that it would not influence the activity of EBF1 in other pathways, such as insulin signaling, or in helping EBF2 to promote thermogenesis in BAT. I am aware that many studies involving anti-inflammatory agents in adipose tissue inflammation have been disappointing [19], but part of the explanation may be that these agents target inflammatory pathways in any or all cells (which could elicit whole-body responses that could easily counter any local metabolic improvement in adipose tissue). Our approach, which involves disabling the APU on the ground, rather than attempting to shut down the engines mid-flight, could go a long way towards prophylactically treating the many metabolic complications of obesity.

## Figures and Tables

**Table 1.** Selected Inflammatory Genes Regulated By Ebf1. Data is from reference [2] and compares the expression of the indicated genes in cells treated with two different sh-Ebf1 constructs, each separately compared to the same “scrambled” control. For instance, expression of *Ccl5* was decreased by 99.2% in cells treated with “shEbf1-A” and by 99.8% in cells treated with “shEbf1-B”. *Ebf1* itself was knocked down by approximately 70% and 75% with the two shEbf1 constructs, respectively.

Gene	Encodes	% of normal expression in EDAs (shEbf1-A)	% of normal expression in EDAs (shEbf1-B)
***Ccl2***	Chemokine (C-C motif) ligand 2 (Monocyte Chemoattractant Protein-1, MCP1)	17.6	21.5
***Ccl5***	Chemokine (C-C motif) ligand 5 (Regulated on Activation, Normal T-Cell Expressed and Secreted, RANTES)	0.8	0.2
***Ccl8***	Chemokine (C-C motif) ligand 8 (Monocyte Chemoattractant Protein-2, MCP2)	22.7	19.1
***Cxcl9***	Chemokine (C-X-C) motif ligand 9	31.0	8.5
***Cxcl10***	Chemokine (C-X-C) motif ligand 10	3.5	0.5
***Il6***	Interleukin-6	58	74
***Tlr2***	Toll-Like Receptor-2	34.3	49.5
***Tlr3***	Toll-Like Receptor-3	35.4	27.6
***Tlr4***	Toll-Like Receptor-4	88 (not significant)	75
***Irf1***	Interferon Regulatory Factor-1	36.3	39.0
***Irf7***	Interferon Regulatory Factor-7	16.5	2.3
***Stat1***	Signal transducer and activator of transcription-1	41.5	19.8
***Myd88***	Myeloid differentiation primary response gene 88	56.4	61.6
